# Mimicking Gene–Environment Interaction of Higher Altitude Dwellers by Intermittent Hypoxia Training: COVID-19 Preventive Strategies

**DOI:** 10.3390/biology12010006

**Published:** 2022-12-21

**Authors:** Rashmi Supriya, Kumar Purnendu Singh, Yang Gao, Dan Tao, Sarah Cheour, Frederic Dutheil, Julien S. Baker

**Affiliations:** 1 Centre for Health and Exercise Science Research, Hong Kong Baptist University, Kowloon Tong, Hong Kong 999077, China; 2 Department of Sport, Physical Education and Health, Hong Kong Baptist University, Kowloon Tong, Hong Kong 999077, China; 3 FEBT, School of Environment, Resources and Development, Asian Institute of Technology, Paholyothin Highway, Klong Luang, Pathum Thani 12120, Thailand; 4 Department of Government and International Studies, Hong Kong Baptist University, Kowloon Tong, Hong Kong 999077, China; 5 High Institute of Sport and Physical Education of Ksar Said, Manouba 2010, Tunisia; 6 University Clermont Auvergne, CNRS, LaPSCo, Physiological and Psychosocial Stress, CHU Clermont-Ferrand, University Hospital of Clermont-Ferrand, Preventive and Occupational Medicine, Witty Fit, F-63000 Clermont-Ferrand, France

**Keywords:** COVID-19, hypoxia, preconditioning, cyclooxygenase, hypoxia training, intermittent hypoxia training

## Abstract

**Simple Summary:**

Cyclooxygenase 2 (COX2) inhibitors have been demonstrated to protect against hypoxia pathogenesis in several investigations. In several studies, it has been shown that COX2 inhibitors guard against the pathogenesis of hypoxia. However, there are conflicting findings on COX inhibitors’ potency in treating COVID-19, and they have drawbacks. As a result, therapeutic COX2 inhibition may not always be beneficial, and further research into potential downstream mediators for hypoxic environment adaptability is required. According to research, those who are used to the lower oxygen levels at altitude may be more resistant to the negative effects of COVID-19. It indicates that COVID-19 poses a greater risk to people who live at lower elevations. It has been demonstrated that the COX2 pathway’s downstream molecules adapt in people who live at high altitudes, which may help to explain why these people have a decreased prevalence of COVID-19 infection. Intermittent hypoxia training can be used to imitate the gene-environment interactions found in people who live at higher altitudes (IHT). It appears that COX-2 adaptation brought on by hypoxia exposure at altitude or IHT serves a significant therapeutic purpose. In this article, we highlight some of the most significant common genes associated with the pathophysiology of COVID-19 and hypoxia. We propose a common pathway between COVID-19 pathogenesis and hypoxia that influences apoptosis, proliferation, the immune system, and metabolism. We also emphasize the importance of researching people who reside at higher elevations to mimic their gene-environment interactions and contrast the results with IHT. Finally, we suggest COX2 as an upstream target for evaluating IHT’s efficacy in preventing or mitigating the consequences of COVID-19 and other oxygen-related pathological conditions in the future.

**Abstract:**

Cyclooxygenase 2 (COX2) inhibitors have been demonstrated to protect against hypoxia pathogenesis in several investigations. It has also been utilized as an adjuvant therapy in the treatment of COVID-19. COX inhibitors, which have previously been shown to be effective in treating previous viral and malarial infections are strong candidates for improving the COVID-19 therapeutic doctrine. However, another COX inhibitor, ibuprofen, is linked to an increase in the angiotensin-converting enzyme 2 (ACE2), which could increase virus susceptibility. Hence, inhibiting COX2 via therapeutics might not always be protective and we need to investigate the downstream molecules that may be involved in hypoxia environment adaptation. Research has discovered that people who are accustomed to reduced oxygen levels at altitude may be protected against the harmful effects of COVID-19. It is important to highlight that the study’s conclusions only applied to those who regularly lived at high altitudes; they did not apply to those who occasionally moved to higher altitudes but still lived at lower altitudes. COVID-19 appears to be more dangerous to individuals residing at lower altitudes. The downstream molecules in the (COX2) pathway have been shown to adapt in high-altitude dwellers, which may partially explain why these individuals have a lower prevalence of COVID-19 infection. More research is needed, however, to directly address COX2 expression in people living at higher altitudes. It is possible to mimic the gene–environment interaction of higher altitude people by intermittent hypoxia training. COX-2 adaptation resulting from hypoxic exposure at altitude or intermittent hypoxia exercise training (IHT) seems to have an important therapeutic function. Swimming, a type of IHT, was found to lower COX-2 protein production, a pro-inflammatory milieu transcription factor, while increasing the anti-inflammatory microenvironment. Furthermore, Intermittent Hypoxia Preconditioning (IHP) has been demonstrated in numerous clinical investigations to enhance patients’ cardiopulmonary function, raise cardiorespiratory fitness, and increase tissues’ and organs’ tolerance to ischemia. Biochemical activities of IHP have also been reported as a feasible application strategy for IHP for the rehabilitation of COVID-19 patients. In this paper, we aim to highlight some of the most relevant shared genes implicated with COVID-19 pathogenesis and hypoxia. We hypothesize that COVID-19 pathogenesis and hypoxia share a similar mechanism that affects apoptosis, proliferation, the immune system, and metabolism. We also highlight the necessity of studying individuals who live at higher altitudes to emulate their gene–environment interactions and compare the findings with IHT. Finally, we propose COX2 as an upstream target for testing the effectiveness of IHT in preventing or minimizing the effects of COVID-19 and other oxygen-related pathological conditions in the future.

## 1. Introduction

Cyclooxygenase (COX) is an enzyme. A class of lipids called prostaglandins is produced at the sites of injury or infection and is used to treat both disease and injury. Prostaglandin G2 intermediates undergo specific conversion to prostaglandin H2 by COX [1]. Many studies have shown that COX2 inhibitors are protective from hypoxic pathogenesis [1], and are being used in COVID-19 treatment as an adjuvant therapy. However, another COX inhibitor, ibuprofen, is linked to an increase in the angiotensin-converting enzyme 2 (ACE2), which could increase virus susceptibility. Hence, inhibiting COX2 via therapeutics might not always be protective and we need to unravel the downstream molecules that may result in hypoxia environmental adaptation.

Interestingly, some of the downstream molecules in the COX2 pathway have been found to be adapted (downregulated)among high-altitude dwellers, which could explain the decreased prevalence of COVID-19 infection. However, more research is needed to directly address COX2 expressions among people living at high altitude. People living at High altitudes (over 8000 feet or 2500 m) provide opportunities to study adaptation and physiological processes [2]. Over the past 25 years, studies on Andeans, Tibetans, and Ethiopians have revealed different oxygen transport characteristics than those of acclimatized newcomers, which may suggest genetic adaptation to high altitude. High altitude short-term (acclimatization, development) and long-term (genetic) responses have a temporal gradient, such that all affect oxygen content, and the long-term (genetic) responses improve blood flow, oxygen delivery, and oxygen metabolism [3].

Recent findings suggest that individuals accustomed to low-oxygen environments at altitude are more likely to cope with COVID-19 and its effects [2]. Researchers suggest that the benefits apply only to individuals who live at high altitudes. Low-altitude travelers still have a higher risk of experiencing severe COVID-19 complications that can be exaggerated by low oxygen levels at higher elevations [4]. COVID-19 impairs normal lung oxygen absorption by affecting the respiratory system leading to a decrease in levels of oxygen in the blood. In a recent review, it was concluded that it is unlikely that individuals traveling to high altitudes would benefit from a lower risk and severity of COVID-19 infection. On the other hand, a high-altitude sojourn does not appear to be associated with additional risks with regards to the pandemic—provided the journey can be made in a safe manner and public health measures are observed [4].

As mentioned previously, low-landers are unlikely to benefit from traveling to higher altitudes. In addition to the lack of benefit, high-altitude exposure may have a negative impact on individual biological equilibriums. Therefore, as an altitude pre-acclimatization strategy, for example, intermittent hypoxic training (IHT) appears to be a promising method to improve immune responses, decrease inflammation, and provide a better toleration of a “silent hypoxemia” state [4]. IHT has also been proposed in previous studies as potential preconditioning for COVID-19 rehabilitation. Previous studies suggest that the application of intermittent hypoxia preconditioning (IHP) on patients may provide inhibitory effects on the levels of various proinflammatory factors and activate hypoxia-inducible factor (HIF-1) to promote target genes to augment erythropoietin (EPO)/vascular endothelial growth factor (VEGF) expression which leads to stimulating the production of the red blood cells, hemoglobin, and angiogenesis to increase the capacity to transport oxygen. Furthermore, activated HIF-1 may mobilize peroxisome proliferator-activated receptor-gamma coactivator-sirtuin 1 (PGC-1-SIRT1)/(AMP-activated protein kinase) AMPK pathway and nitric oxide (NO) availability and inhibit endothelin (ET-1). These factors can help reverse the virus-induced cardiopulmonary hemodynamic disorder and endothelial dysfunction [5]. Many studies point to a lower number and reduced severity of cases in high-altitude cities with decreased oxygen concentrations. Specific literature has shown several benefits of physical training, so, in this sense, physical training using a hypoxic stimulus appears as an alternative that supports the conventional treatments for the COVID-19 patient’s recovery [6]. A clinical study aimed to analyze the effects of moderate-intensity intermittent hypoxic training on health outcomes in COVID-19-recovered patients. Their study provides evidence to support the clinical benefits of moderate IHT as part of the treatment of patients recovered from COVID-19 and also provides evidence on the efficacy and safety of IHT for different health conditions [6].

As part of this paper, we examined the common genes involved in COVID-19 pathogenesis and hypoxia. We propose that hypoxia and COVID-19 pathogenesis share a common mechanism that affects apoptosis, cell proliferation, immunity, and metabolism. In addition, we discuss the importance of mimicking the genes and environmental interactions of individuals living at higher altitudes using IHT. Lastly, we suggest that the effectiveness of IHT in reducing COVID-19 severity or preventing oxygen-related diseases needs to be verified by exploring the expressions of COX2 and its downstream genes after IHT.

## 2. Methodology

The search timeline included studies published until March 2021 and was performed by two researchers using three databases including PubMed, Web of Science, and Google Scholar for this narrative review. The titles/abstracts of the articles were searched using keywords including “COX 2, cyclo-oxygenase, high altitude, adaptation, COVID-19, corona, hypoxia training, intermittent hypoxia training, swimming, yoga, hiking”. The publication language was limited to English. The search included reviews, meta-analyses, systematic reviews, and research articles, and excluded books and book chapters. The search was completed on 1 April 2022. When many similar articles were available, the most recent ones were used. Additional papers were identified from random searches and reference lists of retrieved articles based on our conceptual design. These approaches resulted in a total of 102 articles for possible inclusion within this review.

## 3. Gene-Environment Interaction: A Basic Perspective to Be Targeted for COVID-19

In recent years, there has been increasing interest in finding gene–environment interaction differences in the association of specific genetic variants of disease, or vice versa [7]. It is important to understand these interactions because they can mask the detection of a genetic (or environmental) effect if they are not identified and controlled. They can also cause inconsistencies in the association of diseases when populations are exposed to different environments that modify the effect of a given genetic variant (or vice versa) [8,9,10]. The most important consequence of gene–environment interactions is that they can provide approaches for modifying the effects of deleterious genes by avoiding the corresponding deleterious environmental exposure, since both the genetic variant and the corresponding exposure are needed for disease progression. For example, if a complex disease does not affect people living in a specific environment due to genetic adaptation, then pre-conditioning our bodies and biological systems to adapt to the genetic profiles in those environments may be helpful. This disease avoidance strategy is worthy of future experimentation and investigation.

## 4. High Altitude Decreased COVID-19: A Dilemmatic Implementation

Researchers have discovered that individuals living at high altitudes have a lower susceptibility to coronavirus. One hundred and twenty cities located 3000 m above sea level were compared using COVID-19 data. According to the findings, residents living in high-altitude environments might have advantages against COVID-19 infection [2]. The highlands of various continents (above 2500 m) are home to around 140 million people [11,12]. COVID-19 cases have been documented in Europe, Asia, South America, North America, and Africa’s highlands [13,14,15]. An epidemiological investigation by Arias Reyes et al. (2020) has revealed a decreased reported incidence of COVID-19, implying a possible weak transmission rate of severe SARS-CoV-2 among highland people [16]. Likewise, in the Tibetan Plateau of China, researchers reported a very modest persistent COVID-19 infection [17].

Research has suggested that altitude is associated with COVID-19 mortality among men under 65 years of age. A previous study compared COVID-19 mortality at different altitudes and reported that, since partial oxygen pressure decreases as altitude increases, environmental hypoxia could increase COVID-19 patients’ hypoxemia [18]. Among the seven continents, Asia, South America, and North America have reported that COVID-19 prevalence is lower at higher altitudes than in lower altitudes. Another study used Ecuadorian data to investigate the link between altitude and COVID-19 [19]. Their findings revealed statistically significant differences in incidence, death, and case fatality rates in the Amazon, Sierra, and Costa areas of Ecuador, implying a link between altitude and SARS-CoV-2 transmission and COVID-19 disease severity. Altitude had a 1-unit negative correlation with death rate in univariate analysis [19]. A further study found that COVID-19 prevalence increased across Brazil, a country with continental dimensions, although the disease’s incidence is highly variable, impacting towns and regions differentially [20]. As a result, there is a lack of knowledge relating to the factors that amplify discrepancies in COVID-19 occurrence between Brazilian cities. The researchers explored how altitude affected the occurrence of COVID-19 in Brazilian cities. They examined relative incidence (RI), relative death rate (RDR), and atmospheric relative humidity (RH) of COVID-19 in 154 Brazilian cities with populations above 200 thousand people and elevations between 5 and 1135 m. The association between altitude and RI and RDR, as well as RH and RI and RDR, was investigated using Pearson’s correlation analysis. In the cities studied, they discovered a negative association between COVID-19 incidence and altitude and a positive correlation with RH. They reported COVID-19 in cities with high altitude and low RH have lower RI and RDR. They also proposed that high-altitude cities would be good places to shelter vulnerable people [20]. Furthermore, research also suggests the cause for the decrease in the severity of the global high-altitude COVID-19 outbreak could be related to both environmental and physiological factors, as well as cultural ones; consequently, investigations examining this relationship are needed [21].

## 5. Angiotensin-Converting Enzyme 2 (ACE2) Is the Most Reported Gene for Lower COVID Prevalence in People Living at Higher Elevations

There is currently minimal evidence to suggest any preventive advantage of genetic or non-genetic adaptation to high-altitude hypoxia, including the idea that hypoxia-mediated variations in ACE2 expression or ACE2 variants in certain population groups may be related to illness causation or severity of COVID-19 [22]. Another study compared COVID-19 epidemiology data from Tibet and high-altitude regions of Bolivia and Ecuador to lowland data to examine if high-altitude residents (+2500 m above sea level) are less likely to acquire significant adverse effects from the virus. Based on the current epidemiological evidence, physiological acclimatization/adaptations that counterbalance the hypoxic environment in high-altitude environments may protect against the severe consequences of acute COVID-19 virus infection. Potential underlying mechanisms examined include (i) a virus half-life limited by the high altitude environment, and (ii) hypoxia-mediated decreased expression of ACE2, the virus’s main binding target in the pulmonary epithelium [13]. COVID-19 binds itself to an enzyme called ACE2 to infiltrate cells in the body. ACE2 can be found in a variety of organs, including the heart, lungs, kidneys, and liver. Because individuals become accustomed to living in an environment with less oxygen, persons who have adjusted to living at high altitude generate less ACE2 enzymes. Overall, it is thought that those who reside at higher elevations have a lower risk of contracting COVID-19 because they produce less of the ACE2 enzyme [23]. During our initial search, we observed that, apart from ACE2, very few genes have been studied in people who live at higher altitudes in relation to COVID-19 infection.

## 6. IHT Proposed for Future Prevention of Oxygen-Related Diseases

We suggest that COVID-19 and hypoxia have overlapping pathogenic mechanisms, with COX2 as the shared upstream link. We also reviewed evidence that high-altitude dwellers and lowlanders participating in IHT for the prevention of hypoxia pathogenesis have similar mechanisms in adjusting to hypoxia, with COX2 serving as the common upstream connection.

### 6.1. COVID-19 and Hypoxia Molecules with Similar Mechanisms

#### 6.1.1. Hypoxic Mechanism

Numerous studies have found that COVID-19 or hypoxic environments have comparable mechanisms. COX-2 expression can be increased by hypoxia in a variety of cells, including lung cancer cell lines [24]. The amount of COX-2’s main metabolic product, prostaglandin E2, increases when its expression is deregulated (PGE 2) [25]. PGE 2 generation via the COX-2 catalyzed route is important for hypoxia-inducible factors-1α (HIF-1α) regulation, suggesting that COX-2 inhibitors can prevent hypoxia [26]. COX-2 signaling triggered by COVID-19 has also been suggested to play a function in regulating pulmonary inflammation and injury seen in COVID-19 patients [27]. When tissue demand exceeds oxygen supply, a cascade of intracellular events is triggered, including an increase in the production of hypoxia-inducible factors (HIFs). As a result, an extensive transcriptional response is triggered, which regulates angiogenesis, glucose metabolism, cell proliferation, metastasis, and other activities. The finding of variances between HIF isoforms has revealed new information about the biology of HIFs. Importantly, HIF-1α and HIF-2α can have opposing impacts on the modulation of angiogenic responses. The complexity of the effects exerted by both HIF isoforms as a result of their interaction with other transcription factors should be further investigated, particularly in the context of pro- and anti-angiogenic therapy [28]. HIF-1α, not HIF-2α, appears to control the transcription of genes encoding enzymes that work in a coordinated manner in the glycolytic pathway. A more fundamental question is whether HIF-isoform selectivity has a contribution (if any) in coordinating more complicated patterns of response, such as the dichotomy between pro-survival and proliferative responses to hypoxia and apoptotic and antiproliferative responses to hypoxia [29]. HIF-2α regulates the expression of pro-survival genes such as vascular endothelial growth factor (VEGF), transforming growth factor alpha (TGF-α), and cyclin D1, whereas HIF-1α regulates the expression of proapoptotic genes such as B-cell lymphoma 2 (BCL2)/adenovirus E1B-interacting protein 1, NIP3, and others (BNIP3) [30]. Hypoxia-inducible transcription factors HIF-1α and HIF-2α are also involved in both the hypoxic response and inflammation [31]. In recent years, more attention has been paid to tumor-associated macrophages (TAMs), a distinct macrophage population that expresses M1 products such as interleukin-8 (CXCL8), tumor necrosis factor-alpha (TNF-α), and interleukin-6 (IL-6) as well as M2 compounds such as matrix metalloproteinases (MMPs), interleukin-10 (IL-10), CC chemokine ligand 17 (CCL17) and CC chemokine ligand 22 (CCL22). These variables encourage angiogenesis [32,33,34,35], make tumor cell invasion easier, and/or create an immunosuppressive tumor microenvironment [33,36]. TAMs activity is expected to be complicated and regulated by microenvironmental factors [37]. When macrophages are exposed to hypoxia in vitro, their production of various mitogenic and proangiogenic cytokines changes suggesting that tumor hypoxia has a significant impact on TAM activities [37,38,39,40]. Increased lactate dehydrogenase (LDH) levels are caused by the tumor’s increased glycolytic activity and tumor necrosis caused by hypoxia via HIF-1α [27]. Increases in HIF-2α, on the other hand, promote lipid metabolism [41] and divalent iron absorption [42].

#### 6.1.2. Hypoxia Mechanism in COVID-19

**Figure 1:**
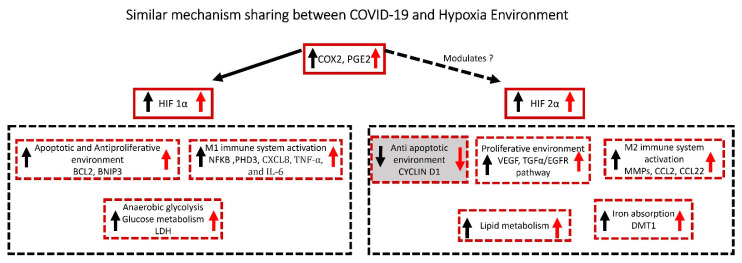
COX2/PGE2 pathway upregulates HIF-1α which in turn increases antiapoptotic and antiproliferative environment (BCL2, BNIP3), increases M1 immune system activation (NFKB, PHD3, CXCL8, TNF-α and IL-6) and supports anaerobic glycolysis as indicated by increase in LDH levels. COX2/PGE2 pathway also modulates HIF-2α that decreases antiapoptotic environment (CYCLIND1), increases proliferative environment (VEGF, TGFα, EGFR) and M2 immune system activation (MMPs, CCL2, CCL22), and supports increases in lipid metabolism and iron absorption (DMT1). Red arrows represent the expressions in people with COVID19 and black arrows represent the expression of molecules under hypoxia environment. COX2 is cyclooxygenase-2, PGE2 is prostaglandin E2, HIF-1α is hypoxia-inducible factor 1-alpha, BCL2 is B-cell lymphoma 2, BNIP3 is BCL2/adenovirus E1B 19 kDa protein-interacting protein 3, NFKB is Nuclear factor kappa B, EGLN1 is egl-9 family hypoxia-inducible factor 1, CXCL8 is C-X-C motif chemokine ligand 8, TNF-α is tumor necrosis factor-alpha, IL-6 is interleukin-6, LDH is lactate dehydrogenase, HIF-2α is hypoxia-inducible factor-2 alpha, (VEGF is vascular endothelial growth factor, TGFα is transforming growth factor alpha, EGFR is epidermal growth factor receptor, MMPs is matrix metalloproteinases, CCL2 and 22 are chemokine (C-C motif) ligand 2 and 22, DMT1 is divalent metal transporter 1.

Similarly, activation of COX2 and HIF-1α in COVID-19 has been shown to upregulate the antiapoptotic antiproliferative microenvironment (BCL2, BNIP3) [43] and M1 immune system {nuclear factor kappa B (NFKB) [44], Egl-9 family hypoxia inducible factor 3 (EGLN3) [45], CXCL8 [46], TNF-α [47], and IL-6 [48]}. Moreover, we propose that COX2 modulates HIF-2α, which in turn downregulates anti-apoptotic microenvironment (cyclin D1) [29,49], increases proliferative microenvironment (VEGF [50], EGFR [51]), and also increases M2 microenvironment compounds, specifically TAMs (e.g., MMPs [52], IL-10 [53], CCL17 [54], and CCL22 [54]), but whether this is through HIF-2α as far as we are aware, is unknown. Increases in antiapoptotic, antiproliferative, and M1 immune system activation occur during increases in anaerobic glycolysis (LDH) [55]. In individuals with viral infections, LDH has been linked to poor outcomes. Elevated LDH levels were linked to a 6-fold increase in the risk of developing severe disease and a 16-fold increase in the risk of death in COVID-19 patients, according to a pooled analysis of 9 published studies (*n* = 1532 COVID-19 patients) [55]. These findings need to be confirmed in larger studies [56]. Increases in lipid metabolism [56] and iron absorption via DMT1 [41] have been documented in COVID-19 individuals, similar to hypoxia. The proposed mechanisms that are shared by hypoxia and COVID-19 are outlined in Figure 1.

#### 6.1.3. High Altitude Adaptation to Hypoxia

Despite numerous studies among high-altitude populations, COX-2 expression has yet to be properly identified. Several studies, however, support the idea that COX-2 plays a critical role in allowing high-altitude dwellers to adapt to hypoxia. Hypoxia, for example, increases COX-2, resulting in the loss of microphthalmia-associated transcription factor (MITF) in cervical stromal cells [57] mediated by prostaglandin E_2_, (PGE2). MITF, which is linked to melanogenesis, and was discovered to be positively selected in Tibetan sheep [58]. Ibuprofen, a COX inhibitor, was found to relieve the symptoms of acute mountain sickness in a meta-analysis [59]. This may make it easier for HIF proteins to be hydroxylated by interacting with EGLN1, causing EGLN1 to be recruited to the HSP90 pathway. Under normoxic conditions, EGLN1 destroys HIF-2α proteins, preventing transcription of HIF-inducible genes such as erythropoietin (EPO), which in turn is responsible for increased red blood cell production [60,61]. Because of HIF-2α’s transcriptional effects on EPO, endothelial PAS domain-containing protein 1 (EPAS1) encodes the oxygen-sensitive a-subunit of the transcription factor HIF-2α, which may also contribute to Tibetans’ blunted erythropoietic response. Finally, HIF-1α transcription factors limit the production of peroxisome proliferator-activated receptor alpha (PPARA) in hypoxic settings, and enhanced PPARA activity is known to lower hemoglobin (Hb) concentration [62]. The most prevalent allelic variants in Tibetans are adversely linked with Hb concentrations in all three of these loci [61,63,64], suggesting that the delayed erythropoietic response in Tibetans may be due to the independent or combined effects of mutations in these genes. Even though this research used diverse genotyping platforms, analytical methodologies, and population samples, they all pointed to the HIF route as the target of natural selection in Tibetan adaptations to high altitude. Despite their comparable evolutionary exposure to high-altitude hypoxia, genome scans in indigenous Andean highlanders (Quechua and Amayra) show little overlap in the genetic targets of selection in Tibetans and Andeans [65,66]. EGLN1, which has positive selection signatures in both populations [61], is a notable exception to this tendency. As a result, apoptotic and antiproliferative environments (BCL2, BNIP3) [67], as well as the M1 immune system microenvironment (NFKB, CXCL8, TNF-α, and IL-6) [68], may be reduced, thereby preventing anaerobic glycolysis (LDH) [69] via HIF-1α suppression. Furthermore, suppression of HIF-2α may result in a reduction or no change in the proliferative environment (VEGF [70], EGFR [71]). However, no changes in TAMs (MMPs, CCL2, CCL22) that is M2 immune system microenvironment have been reported that may play an important role in achieving genetic adaptation and maintaining metabolic homeostasis at high altitude [72]. These changes via HIF-2α may result in adapted lipid metabolism [73] and iron metabolism [63,74].

#### 6.1.4. IHT Mimicking High Altitude Adaptation

**Figure 2:**
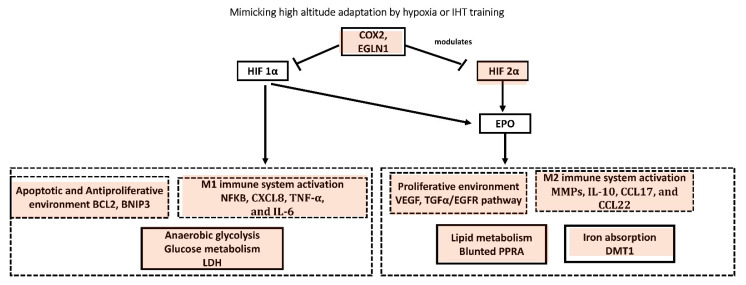
Schematic representation of HIF regulation of erythropoiesis. Gray boxes represent candidate genes that have been hypothesized to contribute to the blunted erythropoietic response and adapted among people living at higher altitude. OX2 is cyclooxygenase-2, PGE2 is prostaglandin E2, HIF-1α is hypoxia-inducible factor 1-alpha, BCL2 is B-cell lymphoma 2, BNIP3 is BCL2/adenovirus E1B 19 kDa protein-interacting protein 3, NFKB is nuclear factor kappa B, EGLN1 is egl-9 family hypoxia-inducible factor 1, CXCL8 is C-X-C motif chemokine ligand 8, TNF-α is tumor necrosis factor-alpha, IL-6 is interleukin-6, LDH is lactate dehydrogenase, HIF-2α is hypoxia-inducible factor-2 alpha, (VEGF is vascular endothelial growth factor, TGFα is transforming growth factor alpha, EGFR is epidermal growth factor receptor, MMPs is matrix metalloproteinases, CCL2 and 22 are chemokine (C-C motif) ligand 2 and 22, DMT1 is divalent metal transporter 1.

Similarly, adapting to hypoxia in the same way as high-altitude individuals would be beneficial by instigating adaptation of similar critical genes that will help individuals deal with hypoxia and oxygen-related disorders. Intermittent hypoxic exposure with exercise training, for example, implies that a brief exposure to hypoxia is adequate to produce HIF-mediated positive muscle adaptations. According to previous studies, leukocytes respond to hypoxia with significant inter-individual variation in HIF-1α mRNA [75]. HIF-1α and HIF-2α mRNA levels are transiently elevated in untrained human skeletal muscle in response to an acute exercise bout, but this reaction is attenuated after exercise training, according to the researchers. They hypothesized that exercise increases HIFs expression, and that it could be a key transcription factor in regulating adaptive gene responses to exercise [76]. According to a narrative review published in 2017, research showed either an increase or no change in hematological variables following altitude training, with factors such as hypoxic dose, training content, athletes’ training background, and/or individual variability of EPO production [77]. Another study looked at the effects of IHT on anaerobic and aerobic capacity as well as swimming performance in well-trained swimmers (*n* = 16). After high-intensity IHT, they found that anaerobic capacity and swimming performance improved significantly. The training program, however, had no effect on absolute VO2max or hematological variables [78]. Another study looked at the impact of hypoxic training on metabolic alterations, notably liver metabolism, in obese mice fed a high-fat diet (HFD). Insulin levels improved after hypoxic training, according to their findings. Furthermore, liver metabolomics revealed information about hypoxia training’s protective effect against HFD-induced fatty liver [79]. Another study investigated the acute inflammatory response to a hypoxia-induced repeat sprint training session. On separate days, 11 amateur team-sport athletes performed a repeat-sprint training (RSH) program (4 sets of sprints) under normoxia and normobaric hypoxic conditions (percentage of inspired oxygen 0.145 to imitate an altitude of 3000 m). When compared to the identical training session performed in normoxia, their findings showed that team-sport athletes can perform an RSH session without increasing inflammation [80]. Another fascinating study looked at the impact of combining yoga with high vitamin D supplementation on the expression and systemic levels of inflammatory cytokines, as well as the psychophysical status of breast cancer survivors [81]. According to their findings, yoga improves physical and psychological fitness and, when combined with a high dose of vitamin D, improves cytokine profiles, which can help manage cancer-related side effects. Another study (*n* = 54) looked at the impact of a resistance exercise program in the presence of intermittent hypoxia on inflammatory biomarkers in seniors. The subjects were randomly assigned to one of three groups: control, resistance training normoxia, and resistance training hypoxia (RTH). The subjects trained under hypoxic conditions at a simulated altitude of 2500 m for 24 weeks. The training program was similar in both experimental groups. Resistance training, whether done in normoxia or hypoxia, was found to be effective in reducing chronic inflammation associated with aging [82]. Another study looked at how endothelial shedding [circulating endothelial cells (ECs)] and hematopoietic stem and progenitor cells (CPCs) changed when 11 healthy unacclimatized participants were exposed to high altitude, after intense exercise, and following an overnight stay in hypobaric hypoxia. MMP activity did not vary throughout the investigation, according to the researchers. MMP-9 concentrations in the blood were linked to EC concentrations, but not MMP activity. Their findings helped researchers learn more about the endothelium and an immature immune system during an active, short-term stay at high altitude [83,84]. The proposed overlapping mechanism mimicking high altitude population and hypoxia or IHT is outlined in Figure 2.

However, we should be aware that hypoxic training will not boost red blood cell production or stroke volume; instead, its effects will occur at a muscular level. Because oxidative pathways are constrained in a hypoxic condition, there is less oxygen available, which reduces endurance performance and forces the body to rely more on anaerobic pathways to generate ATP. It has been demonstrated that training in hypoxia places a larger stress on the body compared to training in normoxia at sea level, resulting in many advantageous adaptations such as better glucose transport, pH regulation, and glycolytic enzymes [85].

### 6.2. The COX Gene as Potential Target to Evaluate the Protective Effects of Hypoxic Training on the Etiology of Respiratory Illnesses

#### 6.2.1. COX as Potential Target for COVID-19

COX genes that are adapted in persons who live at higher altitudes may help them combat COVID-19 more effectively than low-landers. We also demonstrate that these genes have been employed as a therapy for COVID-19 patients to help them cope with the disease. Furthermore, we intend to combine the two concepts because we want to promote the idea that even lowlanders can prepare for the future by slowly adapting their bodies using IHT, such as swimming and yoga, to combat oxygen-related ailments by mimicking highlanders and acclimatizing their COX genes.

COX2 plays an important role in a variety of physiological and pathologic processes. It modulates the expression levels of several serum proteins and plays a key function in viral infections [86]. This enzyme has a large impact on proinflammatory cytokines, but its inhibition or impairment does not prevent the immune system from responding to viral infection. In mice with influenza infection, medicinal inhibition of COX2 by celecoxib reduces TNF-α, granulocyte colony-stimulating factor, and IL-6 levels in bronchoalveolar lavage fluid without a substantial increase in viral titers [87]. COX2 hyperinduction, as well as higher levels of TNF-α and other key proinflammatory cytokines, have been seen in people who suffered from H5N1 infection [88]. Celecoxib, a COX2 inhibitor that is currently on the market, may be able to target several of these essential impacts. These pathophysiologic processes may be particularly essential as the disease progresses from stage 1 to stage 2, when many patients can be treated as outpatients. Celecoxib inhibits p38 mitogen-activated protein kinases (p38MAPK) in addition to COX2, albeit it is not a pure or powerful p38MAPK inhibitor [89]. However, because COX2 inhibition blunts Lipoxin B4-mediated memory B cell activation, it is important to note that COX2 inhibition may result in delayed specific immunoglobulin synthesis [90]. Regardless, the benefits of avoiding a massive cytokine storm appear to outweigh the time it takes to produce specialized antibodies [91]. COX2 inhibition may have antifibrotic effects in COVID-19 patients because epithelial–mesenchymal transition is a key evolutionary phenomenon in many physiologic and disease states of the lung, including lung development, chronic obstructive pulmonary disease (COPD), lung cancer, and pulmonary fibrosis [92,93]. In an animal model, celecoxib also reduced peritoneal fibrosis [94]. Given that interstitial lung fibrosis is one of COVID-19’s signatures, the effects of celecoxib on fibrotic processes may be worth testing clinically, preferably in a randomized controlled trial (RCT) or at the very least off-label under present settings.

#### 6.2.2. COX as Current Adjunct Therapeutic for COVID-19

Representative medications that have previously been shown to be effective in treating SARS-CoV-1, MERS-CoV, HIV, ZIKV, H1N1, and malarial illness offered a strong case for improving the COVID-19 therapeutic doctrine. The efficacy of nonsteroidal anti-inflammatory medications (NSAIDs) such as aspirin, indomethacin, diclofenac, and celecoxib in COVID-19 coagulopathy, as well as their ability to inhibit SARS viral replication, inflammasome deactivation, and synergistic inhibition of H5N1 viral infection with noteworthy antiviral drugs, has provided a bright spot in adjuvant COVID-19 therapy. Because anti-inflammatory NSAIDs and COXIBs work by reversing COX-2 overexpression and modulating the excessive production of pro-inflammatory cytokines and chemokines, they are an effective treatment for COVID-19 infection [95]. COX inhibitors in addition to antiviral medication could help alleviate the vigorous immune response that caused severe respiratory illness; however, they were never explored or studied in RCTs [96,97].

#### 6.2.3. Why Is IHT Training Needed in the Current Era?

Ibuprofen, another COX inhibitor, is linked to an increase in the ACE2 enzyme, which may increase virus susceptibility. Furthermore, as previously stated, inhibiting COX1 may not be a good idea because it reduces antiviral immunity and has little effect on the cytokine storm [98,99]. Celecoxib, another NSAID, has also been evaluated in comparative studies for its cardiovascular side effects. Although some studies demonstrate that celecoxib increases the risk of serious cardiovascular events such as myocardial infarction, heart failure deterioration, and thrombotic cerebral strokes, others show no meaningful difference when compared to more widely prescribed non-selective COX inhibitors. The period of usage and the dose of this medication appear to be two of the most critical characteristics in these investigations [100]. Patients in all these long-term studies received celecoxib for a long time (about 20–30 months), implying that cardiovascular harm is time dependent. However, most of the discussions about producing effective medications turned out to be insignificant, encouraging the discovery of new pathways and pharmaceuticals to combat COVID-19 infection. We propose COX-2 adaptation by hypoxia or IHT may play an important role as it may help in reducing the expression of COX2 without any side effects. Swimming (a form of IHT) was found to reduce the production of pro-inflammatory cytokines and chemokines, as well as the protein expression of phosphorylated NFKBp65 and COX-2, while increasing IL-10 levels. Swimming reduced the production of reactive oxygen species, malondialdehyde, and nitric oxide while increasing glutathione levels, total antioxidant capacity, and the activities of superoxide dismutase and glutathione peroxidase. Swimming also reduced caspase-3 activity and apoptosis-inducing factor, cytochrome c, BCL2-associated X (Bax), and cleaved-caspase-3 expression, while increasing BCL2 levels [101]. Another study found a significant interaction (treatment x treatment duration) effect on the expression profiles of mRNAs for HIF-1α, VEGF, myoglobin, nuclear respiratory factor 1, citrate synthase, carbonic anhydrase 3, monocarboxylate transporter 1, copper/zinc superoxide dismutase, glutathione S-transferase pi, and manganese superoxide dismutase in an obese rat model. Hypoxic living situations, especially when combined with hypoxic exercise training, can result in skeletal muscle health-related biochemical adaptations [102]. We need more research to prove that COX2 is a plausible target for hypoxia or IHT evaluations of their effectiveness in preventing oxygen-related diseases for reducing COVID-19 severity.

## 7. Conclusions and Future Recommendations

We conclude that, in the development of COVID-19 treatments, genetics and environment, or epigenetics, must be investigated further using high-altitude dwellers as an experimental reference. We also recommend that individuals prepare themselves for future oxygen-related diseases. This can be implemented by incorporating hypoxia or IHT into our daily lives, by using exercises such as yoga, swimming, and other high-intensity exercise modalities that will gradually and steadily train our bodies to adapt to low oxygen levels by mimicking high-altitude hypoxic adaptations. In addition, modification of COX2 in hypoxia or IHT needs to be investigated further to examine any therapeutic effects on humans with oxygen-dependent illnesses.

Furthermore, we urge increasing investment in various altitude/hypoxic training facilities including, hypobaric and hypoxic rooms, hotels, masks, training trucks, training facilities, as well as a shift in the awareness, knowledge, and application of altitude/hypoxic training to investigate further the potential health benefits of hypoxia.

## Data Availability

Not applicable.
